# Impact of COVID-19 on Emergency Management of Acute Type A Aortic Dissection: A Single-Center Historic Control Study

**DOI:** 10.31083/j.rcm2306200

**Published:** 2022-05-31

**Authors:** Rui Zhao, Wei Xu, Zhao Wang, Cuntao Yu, Yanmin Yang

**Affiliations:** ^1^Department of Cardiovascular Surgery, Fuwai Hospital, National Clinical Research Center for Cardiovascular Diseases, National Center for Cardiovascular Diseases, Chinese Academy of Medical Sciences and Peking Union Medical College, 100037 Beijing, China; ^2^Emergency Center, Fuwai Hospital, National Clinical Research Center for Cardiovascular Diseases, National Center for Cardiovascular Diseases, Chinese Academy of Medical Sciences and Peking Union Medical College, 100037 Beijing, China; ^3^Department of Cardiology, Fuwai Hospital, National Clinical Research Center for Cardiovascular Diseases, National Center for Cardiovascular Diseases, Chinese Academy of Medical Sciences and Peking Union Medical College, 100037 Beijing, China

**Keywords:** COVID-19, emergencies, aortic dissection, epidemics

## Abstract

**Background:**

The present study aimed to clarify the impact of the 2020 COVID-19 pandemic on emergency management of acute type A aortic dissection.

**Methods:**

We consecutively enrolled 337 acute type A aortic dissection (ATAAD) patients at emergency room in Fuwai Hospital (Beijing, China) from January to June during the 2020 COVID-19 epidemic (n = 148) and the same period in 2019 as the historical control (n = 189). The primary outcome was defined as in-hospital death. Other outcomes included automatic discharge during emergency admission. The factors with significant differences before and after the epidemic were compared and analyzed by stages with the study endpoint to clarify their changes in different stages of the epidemic.

**Results:**

There was no significant difference in in-hospital mortality (35 (20.5%) vs. 23 (17.4%), *p* = 0.472). Compared with year 2019, proportion of patients receiving surgical treatment decreased significantly (74 (50.0%) vs. 129 (68.25%), *p <* 0.001). The surgery time of ATAAD patients in 2020 was significantly shorter (6.46 [5.52, 7.51] vs. 7.33 [6.00, 8.85] hours, *p* = 0.01). The length of stay in the emergency department significantly differed at each stage.

**Conclusions:**

Our study demonstrated a significant reduction in the number of ATAAD patients and surgical treatment during COVID-19 outbreak. The surgical strategy of patients changed, but the overall mortality was largely the same. Patients undergoing surgery had a trend toward longer interval from the onset to the operating room, but they tended to be normal at the end of the epidemic. Proper epidemic prevention policies may avoid COVID-19 hitting patients who are not infected with the virus to the greatest extent.

## 1. Introduction

As of February 2022, the ongoing COVID-19 epidemic has caused more than 5.7 million deaths worldwide [1]. In the past two years, COVID-19 has not been well controlled, and health care resources remain strained especially for critically ill patients. Acute type A aortic dissection (ATAAD) is an emergent disease and requires timely surgical treatment [2]. The coexistence of COVID-19 and ATAAD is usually a fatal disaster [3]. The ongoing COVID-19 crisis has had a significant impact on ATAAD management worldwide. The number of surgical cases for ATAAD per month decreased significantly and sharply in New York during the COVID-19 epidemic [4]. Arnaud *et al*. [5] hypothesized that this reduction in the number of ATAAD patients was inaccurate, largely because many patients avoid participating in medical counseling, thereby preventing them from receiving needed health care. Delayed treatment has an important impact on the management of type A aortic dissection due to infection and transmission associated with the COVID-19 epidemic. Moreover, some studies only showed the surgical results of ATAAD during the COVID-19 epidemic, and did not consider the impact of delayed treatment [6]. The present study aimed to clarify the impact of the COVID-19 epidemic on the emergency management of acute type A aortic dissection.

## 2. Methods

### 2.1 Study Population

We performed a single-center historic control study, and the clinical data, surgical characteristics and in-hospital outcomes were collected from Electronic Medical Records. We enrolled all the ATAAD patients without aortic intramural hematoma at the emergency room from January 1st to June 30th during the COVID-19 epidemic in Fuwai Hospital. We also included all ATAAD patients who were treated in Fuwai Hospital from January 1st and June 30th in 2019 as historical controls. This study is in line with the Declaration of Helsinki. The ethics committee approved the study protocol, and the institutional review board waived the requirement for obtaining informed consent because the data were acquired for routine patient care and all data used for this study were acquired for clinical purposes and processed anonymously.

### 2.2 Treatment Principles for ATAAD During the COVID-19 Epidemic

In our institution, all ATAAD patients should complete an epidemiological history survey. Excluding COVID-19 contact history, the new coronavirus RT-PCR tests and blood samples for COVID-19 specific IgG and IgM were collected, and lung CT scans were arranged in the emergency room. In the meantime, drugs were used to control patients’ blood pressure and heart rates. If all of these above results above were negative, the ATAAD patient was immediately arrange for surgery. Patients in whom COVID-19 could not be ruled out temporarily, defined as the absence of epidemiological history of COVID-19, with 1–2 clinical manifestations of COVID-19 but not fulfilling the diagnostic criteria for COVID-19, were transferred to designated clinics and treated with medical therapy. At the same time, patients were screened for COVID-19 and transferred to designated hospitals if test was positive. Patients with confirmed or suspected COVID-19 according to the COVID-19 Diagnosis and Treatment (7th edition) were transferred to COVID-19-designated hospitals and received medical therapy as soon as possible [7].

### 2.3 Clinical Variables Definition

The primary outcome was defined as in-hospital death. Other outcomes included automatic discharge during emergency admission. Automatic discharge was defined as refusal to accept surgical treatment and medical treatment, but the patients’ vital signs were stable at discharge. The transport distance of the patient was defined as the driving distance from the patient’s current residential address to our center, which was calculated using Alibaba cloud and Auto Navi Map (https://lbs.amap.com/). The onset time was defined as the time between onset of the first symptoms to arrival in the emergency room. The emergency room stay time was defined as the time from emergency department admission to surgery. The interval from onset to operation was defined as the sum of onset time and emergency stay time. According to the Fighting COVID-19: China in Action published by the State Council Information Office of the People’s Republic of China, the COVID-19 epidemic was divided into 5 stages. Stage I: Swift Response to the Public Health Emergency (December 27, 2019–January 19, 2020); Stage II: Initial Progress in Containing the Virus (January 20–February 20, 2020); Stage III: Newly Confirmed Domestic Cases on the Chinese Mainland Drop to Single Digits (February 21–March 17, 2020); Stage IV: Wuhan and Hubei – An Initial Victory in a Critical Battle (March 18–April 28, 2020); Stage V: Ongoing Prevention and Control (Since April 29, 2020) [8].

### 2.4 Statistical Analysis

Continuous data are expressed as means ± SD or medians (interquartile range, IQR) and categorical variables are presented as the counts and percentages. The continuous variables were compared using Student’s test or the Mann-Whitney test as appropriate. The categorical variables were compared by the Chi-square test or Fisher exact test as appropriate. A *p *value less than 0.05 was considered significant. The factors with significant differences before and after the epidemic were compared and analyzed by stages with the study endpoint to clarify their changes in different stages of the epidemic. All analyses were performed by R (version 4.1.0., https://www.r-project.org).

## 3. Results

### 3.1 Baseline and Clinical Characteristics of ATAAD Patients

**Fig. 1.**
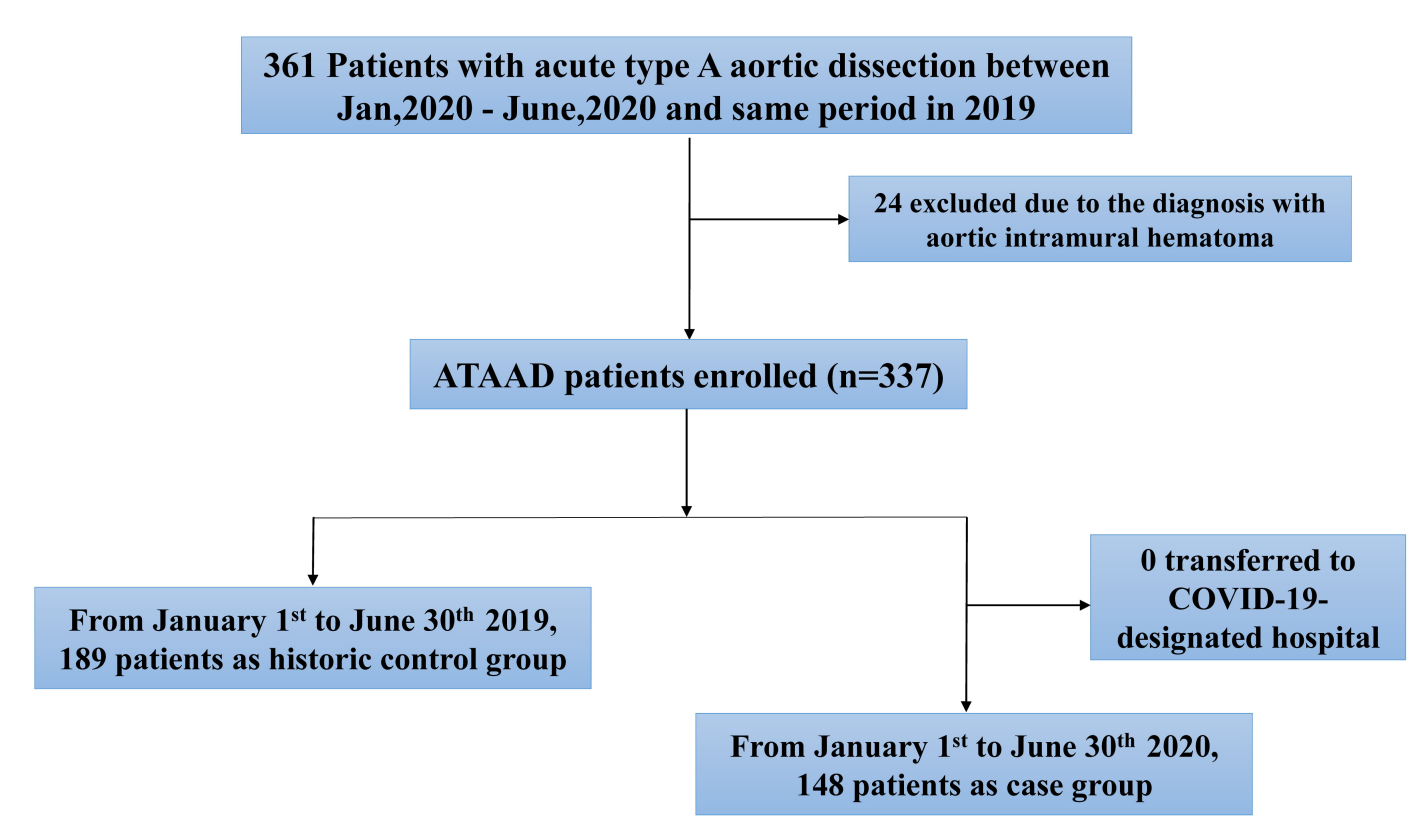
**Patient selection diagram**. A total of 337 patients with ATAAD were enrolled in this study.

**Fig. 2.**
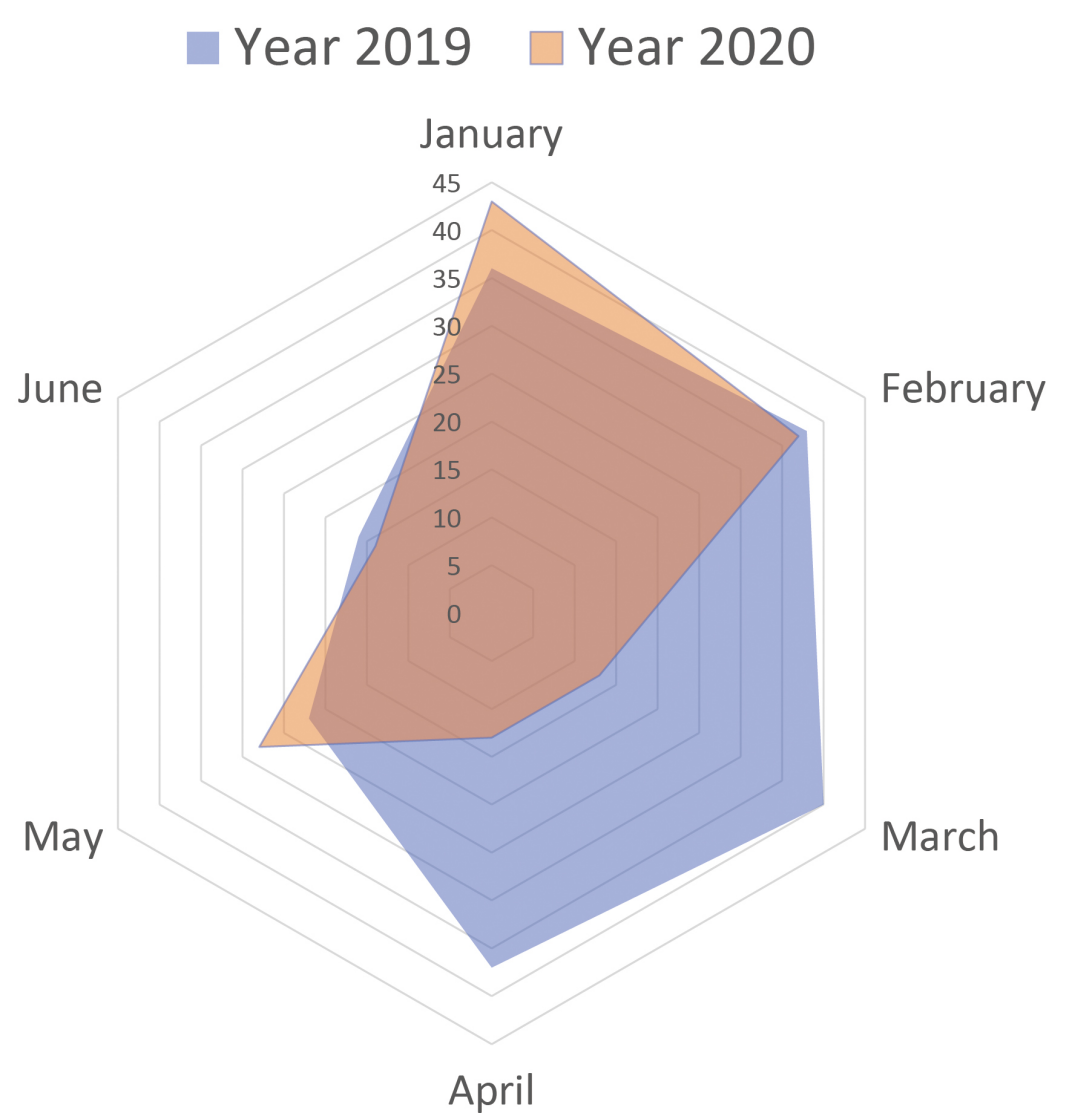
**Comparison of the number of patients admitted**. The radar chart showed the number of ATAAD emergency admission in each natural month in the first half of 2019/2020.

**Fig. 3.**
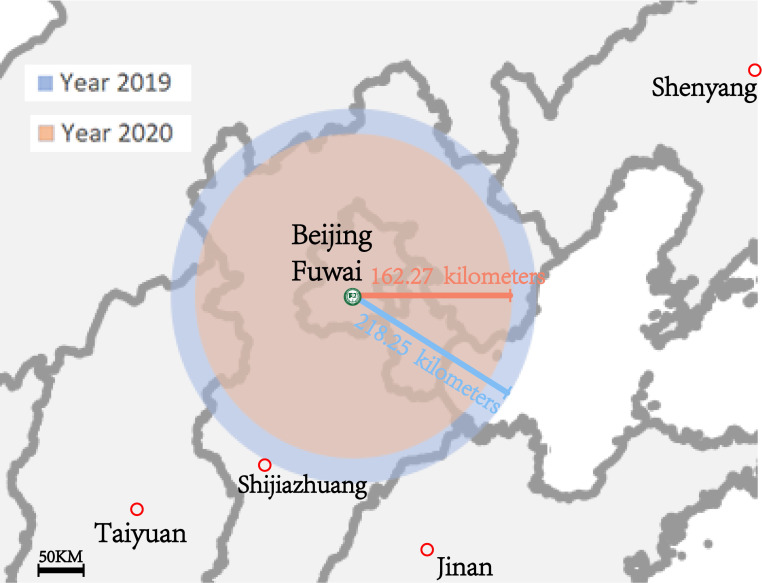
**Impact of COVID-19 pandemic on patient median transport distance on the map**. Compared with 2019, patients come from areas closer to the hospital.

Overall, 337 patients with ATAAD were enrolled in this study and no patients were transferred to COVID-19-designated hospital (Fig. 1). During the COVID-19 epidemic in China from January 1st to June 30th, 2020, a total of 148 consecutive patients entered the emergency department diagnosed with ATAAD at Fuwai Hospital. During the same period in 2019, a total of 189 consecutive ATAAD patients were admitted to the emergency department (Fig. 2). There was no significant difference in the median time of onset before and after the COVID-19 epidemic (13 [7, 30] hours vs. 12 [6, 33] hours, *p* = 0.554). The median emergency stay time and interval from the onset to the operating room was significantly prolonged among patients who received surgery (16.75 [10.83, 25.65] hours vs. 26.75 [15.20, 45.87] hours, *p *< 0.001; 38.42 [22.92, 81.25] hours vs. 47.12 [29.48, 96.06] hours, *p* = 0.049). After the outbreak of the epidemic, the median transport distance from the residence to the medical center was shortened, the difference was not significant (218.25 [65.86, 484.08] vs. 162.27 [47.94, 436.87] kilometers, *p* = 0.656) (Fig. 3). There were no significant differences in baseline and clinical characteristics of the total study population between groups (Table 1).

**Table 1.** **Baseline and clinical characteristics of ATAAD patients according to the year of hospitalization**.

	Year 2019	Year 2020	*p *value
	N = 189	N = 148	
Age (mean (SD)), years	53.68 (12.67)	55.55 (14.18)	0.202
Male, n (%)	131 (69.3)	110 (74.3)	0.373
BMI (mean (SD)), kg/$m^{2}$	26.36 (3.77)	27.10 (4.69)	0.247
Transport distance (median [IQR]), km	218.25 [65.86, 484.08]	162.27 [47.94, 436.87]	0.656
Onset time (median [IQR]), h	13.00 [7.00, 30.00]	12.00 [6.00, 33.00]	0.554
Emergency stay time (median [IQR]), h	16.75 [10.83, 25.65]	26.75 [15.20, 45.87]	<0.001
Interval from onset to operation (median [IQR]), h	38.42 [22.92, 81.25]	47.12 [29.48, 96.06]	0.049
Pain, n (%)	180 (95.2)	133 (91.7)	0.278
Chest pain, n (%)	130 (68.8)	109 (75.7)	0.278
Back pain, n (%)	81 (42.9)	58 (40.0)	0.278
Abdominal pain, n (%)	42 (22.2)	22 (15.2)	0.278
Syncope, n (%)	20 (10.6)	9 (6.2)	0.226
Coma, n (%)	5 (2.6)	3 (2.1)	1
Tamponade, n (%)	6 (3.2)	6 (4.1)	0.882
Hypertension, n (%)	156 (83.4)	122 (82.4)	0.926
Diabetes, n (%)	12 (6.5)	8 (5.5)	0.881
Coronary heart disease, n (%)	20 (10.9)	24 (16.7)	0.172
COPD, n (%)	3 (1.6)	0 (0.0)	0.341
Chronic kidney disease, n (%)	3 (1.6)	1 (0.7)	0.792
Marfan syndrome, n (%)	3 (1.6)	1 (0.7)	0.797
Bicuspid aortic valve, n (%)	8 (4.3)	2 (1.4)	0.223
Previous aortic surgery history, n (%)	5 (2.7)	4 (2.7)	1
Previous cardiac surgery, n (%)	5 (2.7)	3 (2.0)	0.98
cTnI (median [IQR]), μg/L	0.00 [0.00, 0.02]	0.01 [0.00, 0.03]	0.387
NT proBNP (median [IQR]), ng/L	220.10 [81.88, 608.80]	239.90 [80.38, 805.70]	0.958
Hemoglobin (median [IQR]), g/L	136.00 [124.00, 148.00]	143.00 [132.25, 151.75]	0.02
White blood cell (median [IQR]), *${\mathrm{10}}^{9}$/L	10.94 [9.37, 13.68]	11.80 [9.53, 14.83]	0.25
Platelet (median [IQR]), *${\mathrm{10}}^{9}$/L	177.00 [150.00, 214.00]	185.00 [156.25, 219.00]	0.448
Creatinine (median [IQR]), μmoI/L	88.70 [74.30, 106.60]	84.62 [76.32, 105.04]	0.773
D-Dimer (median [IQR]), mg/L	7.60 [2.01, 18.51]	7.17 [2.78, 13.92]	0.76
Aortic insufficiency, n (%)			0.42
none	53 (29.3)	36 (25.2)	
mild	66 (36.5)	50 (35.0)	
moderate	56 (30.9)	47 (32.9)	
severe	6 (3.3)	10 (7.0)	
Ejection fraction (median [IQR]), %	60.00 [58.00, 63.00]	60.00 [58.00, 62.00]	0.714

BMI, body mass index; COPD, chronic obstructive pulmonary disease.

### 3.2 Clinical Outcomes According to the Year of Hospitalization

**Fig. 4.**
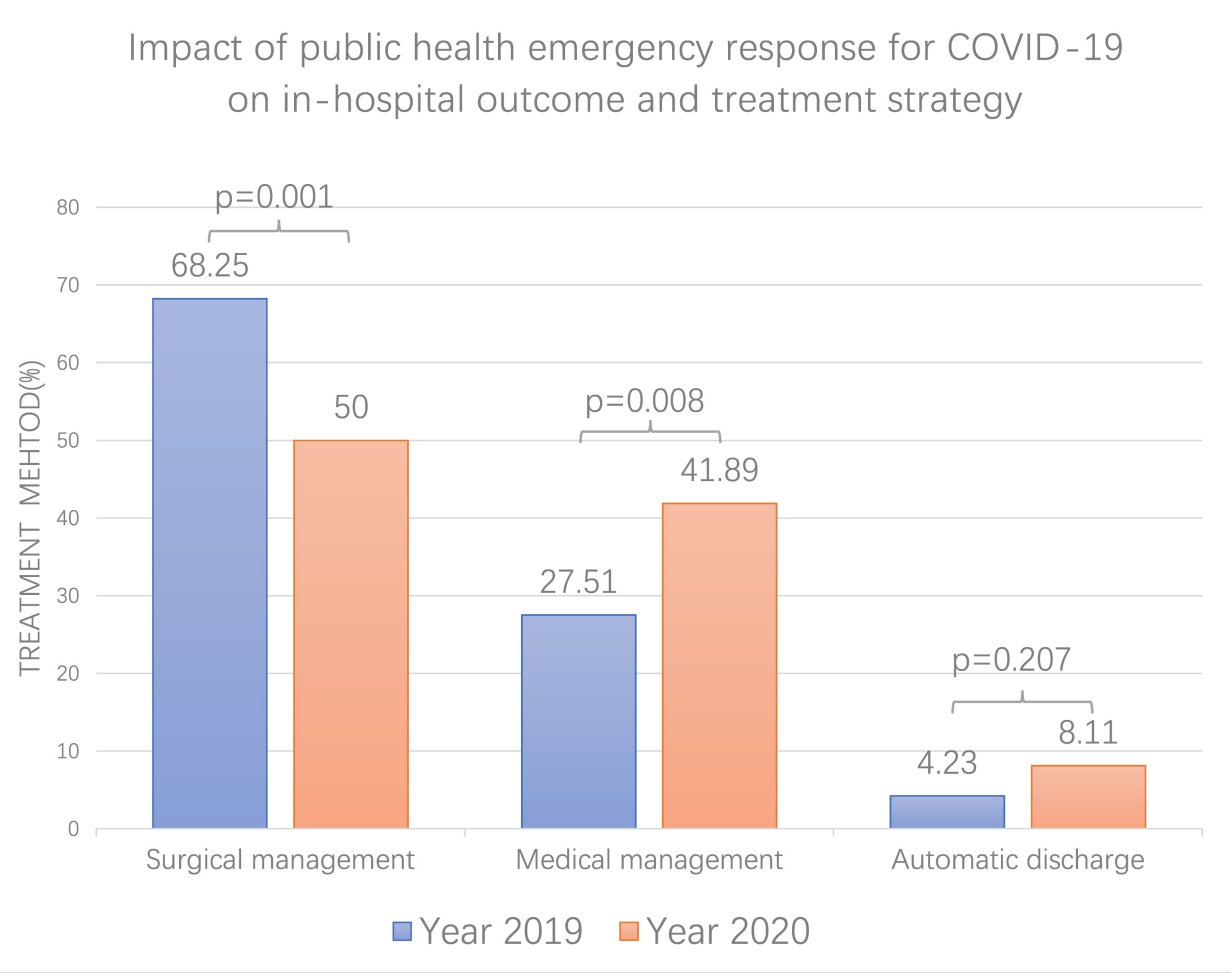
**Impact of COVID-19 pandemic on treatment strategy of ATAAD**. The proportion of patients receiving surgical treatment during the epidemic decreased significantly and patients receiving medical treatment increased significantly

The clinical outcomes of the two groups were also compared (Table 2). There was no significant difference in in-hospital mortality (35 (20.5%) vs. 23 (17.4%), *p* = 0.472). Although the proportion of automatic discharge in 2020 is close to twice that in 2019, there was still no significant difference (12 (8.1%) vs. 8 (4.2%), *p* = 0.207). The proportion of patients receiving surgical treatment during the epidemic decreased significantly (74 (50.0%) vs. 129 (68.25%), *p *< 0.001) and patients receiving medical treatment increased significantly (52 (27.5%) vs. 62 (41.9%), *p* = 0.008) (Fig. 4).

**Table 2.** **Clinical outcomes according to the year of hospitalization**.

	Year 2019	Year 2020	*p* value
	N = 189	N = 148	
In hospital death, n (%)	35 (20.5)	23 (17.4)	0.472
Surgical management death, n (%)	8 (6.2)	1 (1.5)	0.094
Medical treatment death, n (%)	27 (14.3)	22 (14.9)	0.881
Automatic discharge at emergency room, n (%)	8 (4.2)	12 (8.1)	0.135

### 3.3 Surgical Management and Postoperative Complications

Compared with 2019, the surgery time of ATAAD patients was significantly shorter in 2020 (6.46 [5.52, 7.51] vs. 7.33 [6.00, 8.85] hours, *p* = 0.01), but there were no significant differences in cardiopulmonary bypass time (178.00 [145.50, 212.50] vs. 183.00 [150.75, 242.00] minutes, *p* = 0.411), aortic cross clamping time (118.00 [95.00, 149.00] vs. 114.50 [91.00, 145.00] minutes, *p *= 0.71) or circulatory hypothermic arrest time (15.00 [9.00, 18.00] vs. 15.50 [2.25, 20.00] minutes, *p* = 0.836). In 2020, patients were treated with more extended and more complex operations (David procedure: 4 (5.9%) vs. 1 (0.8%), *p* = 0.093; total arch replacement: (59 (86.8%) vs. 109 (85.2%), *p* = 0.927), but there were no significant differences except the increase in the proportion of frozen elephant trunk implantation (52 (76.5%) vs. 78 (60.9%), *p* = 0.042). This also indicates that the proportion of total aortic arch replacement using hybrid technology is reduced (7 (10.3%) vs. 26 (20.3%), *p *= 0.113). There were no significant differences in other surgical methods or postoperative complications between groups (Table 3).

**Table 3.** **Surgical management and postoperative complications according to the year of hospitalization**.

	Year 2019	Year 2020	*p *value
	N = 129	N = 74	
Postoperative death, n (%)	8 (6.2)	1 (1.5)	0.245
Surgery time (median [IQR]), h	7.33 [6.00, 8.85]	6.46 [5.52, 7.51]	0.01
CPB time (median [IQR]), min	183.00 [150.75, 242.00]	178.00 [145.50, 212.50]	0.411
ACC time (median [IQR]), min	114.50 [91.00, 145.00]	118.00 [95.00, 149.00]	0.71
HCA time (median [IQR]), min	15.50 [2.25, 20.00]	15.00 [9.00, 18.00]	0.836
Ascending replacement only, n (%)	3 (2.3)	0 (0.0)	0.509
Bentall procedure, n (%)	30 (23.4)	17 (25.0)	0.946
David procedure, n (%)	1 (0.8)	4 (5.9)	0.093
Wheat’s procedure, n (%)	3 (2.3)	4 (5.9)	0.386
Partial Arch Replacement, n (%)	6 (4.7)	8 (11.8)	0.124
Total Arch Replacement, n (%)	109 (85.2)	59 (86.8)	0.927
Hybrid Arch Replacement, n (%)	26 (20.3)	7 (10.3)	0.113
Frozen Elephant Trunk, n (%)	78 (60.9)	52 (76.5)	0.042
Coronary Artery Bypass Graft, n (%)	26 (20.3)	13 (19.1)	0.991
Blood loss (median [IQR]), mL	705.00 [600.00, 900.00]	810.00 [622.50, 900.00]	0.644
Red blood cell input (median [IQR]), u	0.00 [0.00, 3.50]	0.00 [0.00, 2.00]	0.378
Plasma input (median [IQR])	400.00 [0.00, 600.00]	400.00 [100.00, 600.00]	0.914
Platelet input (median [IQR])	1.00 [1.00, 1.00]	1.00 [1.00, 1.00]	0.377
Mechanical ventilation time (median [IQR])	18.00 [13.00, 56.75]	19.00 [14.00, 39.25]	0.937
Readmission ICU, n (%)	4 (3.1)	0 (0.0)	0.343
Re exploration for bleeding, n (%)	2 (1.6)	0 (0.0)	0.768
Sternal wound infection, n (%)	2 (1.6)	0 (0.0)	0.772
Pneumonia, n (%)	49 (38.3)	35 (51.5)	0.104
Tracheotomy, n (%)	2 (1.6)	0 (0.0)	0.772
Respiratory failure, n (%)	6 (4.7)	4 (5.9)	0.983
Pleural effusion, n (%)	7 (5.5)	3 (4.4)	1
Pericardial effusion, n (%)	4 (3.1)	0 (0.0)	0.346
Gastrointestinal bleeding, n (%)	2 (1.6)	0 (0.0)	0.772
Acute kidney insufficiency, n (%)	24 (18.8)	12 (17.6)	1
CRRT, n (%)	12 (9.4)	2 (2.9)	0.17
Stroke, n (%)	5 (3.9)	1 (1.5)	0.612
Mental symptoms, n (%)	14 (10.9)	14 (20.6)	0.104
Paraplegia, n (%)	3 (2.3)	2 (2.9)	1
MODS, n (%)	0 (0.0)	1 (1.5)	0.747
ECMO, n (%)	0 (0.0)	1 (1.5)	0.747
IABP, n (%)	0 (0.0)	1 (1.5)	0.747

CPB, cardiopulmonary bypass; ACC, aortic cross-clamping; HCA, hypothermic circulatory arrest; CRRT, continuous renal replacement therapy; MODS, multiple organ dysfunction syndrome; ECMO, extracorporeal membrane oxygenation; IABP, intra-aortic balloon pump.

### 3.4 Dynamic Changes of Clinical Characteristics of ATAAD Patients during COVID-19

**Fig. 5.**
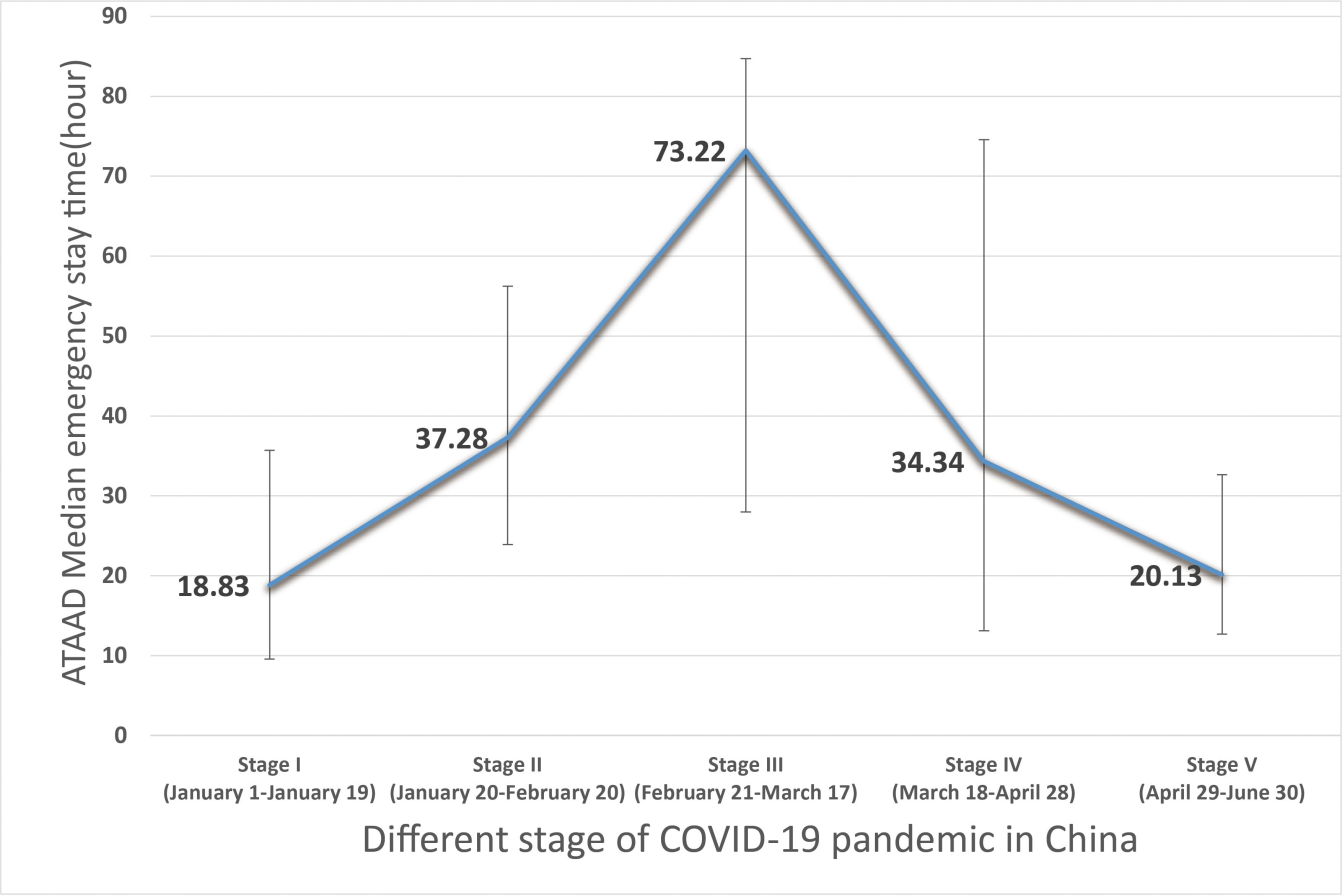
**ATAAD median emergency stay time according to different stage of 2020 COVID-19 pandemic in China**. COVID-19 has limited impact on emergency ATAAD patients.

We analyzed the treatment options, in-hospital mortality, proportion of automatic discharge, onset time, emergency stay time, and the interval from onset to operation according to the epidemic stage during 2020 (Table 4). Among these factors, only the emergency stay time in each stage showed significant differences. In stage III of the COVID-19 epidemic, the interval was the longest (73.22 [28.00, 84.73] hours), while in the stage V, it was almost the same as that in the stage I and that in year 2019 (20.13 [12.71, 32.66] vs. 18.83 [9.57, 35.70] vs. 16.75 [10.83, 25.65] hours) (Fig. 5).

**Table 4.** **Main clinical characteristics according to different stage of COVID-19 pandemic during the year 2020**.

	Stage I	Stage II	Stage III	Stage IV	Stage V
n	N = 25	N = 49	N = 13	N = 18	N = 43
Surgical management, n (%)	12 (48.0)	26 (53.1)	7 (53.8)	8 (44.4)	21 (48.8)
Medical management, n (%)	9 (36.0)	17 (34.7)	6 (46.2)	9 (50.0)	21 (48.8)
Automatic discharge, n (%)	4 (16.0)	6 (12.2)	0 (0.0)	1 (5.6)	1 (2.4)
Emergency death, n (%)	1 (4.0)	8 (16.3)	2 (15.4)	3 (16.7)	8 (18.6)
Surgical death, n (%)	0 (0.0)	1 (4.2)	0 (0.0)	0 (0.0)	0 (0.0)
Onset time (median [IQR]), h	18.00 [6.50, 48.00]	13.00 [8.00, 40.00]	11.00 [5.00, 24.00]	9.00 [5.00, 24.00]	12.00 [7.00, 24.00]
Emergency stay time (median [IQR]), h	18.83 [9.57, 35.70]	37.28 [23.92, 56.23]	73.22 [28.00, 84.73]	34.34 [13.12, 74.56]	20.13 [12.71, 32.66]
Interval from onset to operation (median [IQR]), h	29.91 [22.90, 57.48]	69.66 [44.19, 103.37]	79.33 [73.46, 145.49]	36.46 [28.50,108.19]	39.78 [25.67, 96.17]

## 4. Discussion

The study reviewed the impact of the COVID-19 epidemic on emergency management of type A aortic dissection. Our results showed a significant reduction in the number of ATAAD patients and surgical treatment during the COVID-19 outbreak. Correspondingly, the proportion of conservative medical treatment increased. Patients enrolled during the COVID-19 epidemic undergoing surgery had a trend toward longer median emergency stay times and intervals from the onset to the operating room, but they tended to be normal at the end of the epidemic. In addition, a larger proportion of these patients received an open rather than hybrid surgical strategy, but the overall mortality did not largely change compared with the historic controls.

Our study showed a 21% reduction in emergency admissions for ATAAD. Similarly, during the same period, the number of emergency admissions for ST-segment elevation myocardial infarction (STEMI) in our center decreased by 23.2% [7]. Some studies showed that the frequency of seeking medical advice in some patients with cardiovascular disease was lower than normal during the COVID-19 epidemic [4, 9, 10, 11]. However, the patient transport distance in our study was shortened by a quarter after the COVID-19 outbreak. These patients might not have chosen to transfer to our center but chose to receive treatment in the local medical center. It is worth noting that a study from Michigan showed that the number of ATAAD operations did not decrease between 2019 and 2020 [12]. Therefore, the incidence ATAAD may not have decreased during the epidemic, but the referral mode may change. In communication with provincial hospitals around Beijing, they confirmed that local ATAAD patients increased during the epidemic, but it was not reported in international journals. Multicenter research with nearby provincial medical centers should be conducted to further clarify this conclusion.

COVID-19 is highly infectious, and many patients and carriers have only mild atypical symptoms, which poses challenges for prompt diagnosis and treatment [13]. A previous study reported that one ATAAD patient initially underwent surgery without being suspected of COVID-19. However, this patient later tested positive, and two medical staff involved with that patient subsequently tested positive [12]. Therefore, it is very important to identify these patients infected with COVID-19 in the emergency room, which can prevent large-scale spread of virus in hospitals and further threaten the health of medical staff and other patients. Many scholars strongly advocate mandatory testing, regardless of hemodynamic instability or the presence of suspected COVID-19 [6, 12, 14]. However, at the early stage of the epidemic, RT-PCR was unavailable for the detection of COVID-19, and chest computerized tomography (CT) may be considered a primary tool for COVID-19 detection in epidemic areas [15]. However, in the presence of ATAAD, the positive predictive value of CT scans for COVID-19 is low. Because typical imaging features of COVID-19 pneumonia are very similar to those of ATAAD in the lungs [12]. In addition, the RT-PCR usually took longer time at the very beginning of the epidemic, which is also one of the main reasons for increased emergency stay time. However, in stage V of the epidemic, the medical center increased its efficiency of RT-PCR detection, and the detection time was shortened to less than two hours. This result was also confirmed in our study by the dynamic evolution of emergency stay time, which shows that the impact of the COVID-19 epidemic on emergency management of type A aortic dissection only lasted a limited time. Proper epidemic prevention policy also avoids COVID-19 hitting patients who are not infected with the virus to the greatest extent.

It is undeniable that urgent surgery is still the primary treatment of ATAAD [2]. However, in our study, the proportion of ATAAD patients receiving surgical treatment decreased compared with that before the COVID-19 epidemic. This may have been caused by delayed surgery during the epidemic. In some patients, chest pain and other symptoms disappeared during continuous medical treatment. They chose to leave the hospital, and these patients became chronic type A aortic dissection. Research from Switzerland showed that delayed treatment of non– COVID-related diseases caused by the COVID-19 epidemic had a significant impact on patient safety [5]. We maybe witnessing an increase of chronic type A aortic dissection as a collateral effect of the COVID-19 epidemic. Fortunately, in our previous study, the surgical treatment of chronic type A aortic dissection was not a threat and current surgical strategies for ATAAD were applicable to chronic TAAD with excellent outcomes [16]. Although the waiting strategy for ATAAD is controversial, aggressive medical management can ensure safety. Many patients with type A aortic dissection can be safely managed nonoperatively short-term at experienced aortic centers. More importantly, even surgery in elderly and/or severely complicated patients cannot change the fatal results, and it may also increase the COVID-19 exposure of medical staff and in-hospital patients [12].

ATAAD can be treated with a variety of surgical strategies [17, 18]. In our center, we prefer total arch replacement combined with frozen elephant trunk surgery to hybrid surgery during the epidemic. This is because the number of hybrid operation rooms is less than that of conventional operation rooms. To receive surgical treatment as soon as possible, we arrange patients in conventional operation rooms instead of waiting for hybrid operation rooms. In another heart center in Beijing, the proportion of FET procedure in the same period was 72%, which is similar to our results [6]. There were no significant difference in survival between FET and hybrid surgery [19]. In our study, the postoperative outcomes including the incidence of complications also confirmed this result. In addition, some patients with subacute stage who have passed the acute stage are relatively stable. Previous studies have shown that these patients experienced shorter operation times [16]. These changes reflect how necessary it is to flexibly select appropriate surgical strategies during the COVID-19 epidemic.

This retrospective study has several limitations. For some patients discharged automatically, we did not know their long-term prognosis, and our emergency room did not have the contact information of these patients. As the largest cardiovascular center in China, the situation of our patients is not very complicated. In our cohort, none of the patients had COVID-19 infection. Although the situation in our center is quite unique, we hope that our results will inspire other centers in the face of COVID-19, including the recent epidemic of the Omicron variant.

## 5. Conclusions

Our study demonstrated a significant reduction in the number of ATAAD patients and surgical treatment during the COVID-19 outbreak. The surgical strategy of patients changed, but the overall mortality was largely same. Patients undergoing surgery had a trend toward longer intervals from the onset to the operating room, but they tended to be normal at the end of the epidemic. Proper epidemic prevention policy may avoid COVID-19 hitting patients who are not infected with the virus to the greatest extent.
